# Neuromodulation effects of low-intensity transcranial focused ultrasound in human, a systematic review focusing on motor and sensory functions

**DOI:** 10.1186/s12984-025-01722-9

**Published:** 2025-11-28

**Authors:** Heng-Chen Ho, Wen-Shiang Chen, Ming-Yen Hsiao

**Affiliations:** 1 Department of Physical Medicine and Rehabilitation, College of Medicine, National Taiwan University, No.7, Zhongshan S. Rd., Zhongzheng Dist, Taipei, 100 Taiwan; 2 Department of Physical Medicine and Rehabilitation, National Taiwan University Hospital, Taipei, Taiwan

**Keywords:** Transcranial focused ultrasound, Neuromodulation, Non-invasive brain stimulation, Motor function, Sensory function

## Abstract

**Background:**

Low-intensity transcranial focused ultrasound (tFUS) has emerged as a novel non-invasive brain stimulation technique with therapeutic potential for various neurological conditions. However, its parameter-dependent bimodal neuromodulatory effects remain to be fully elucidated.

**Objective:**

This study aims to review the evidence on tFUS-induced neuromodulation in humans, particularly its effects on motor and sensory functions, to identify specific stimulation parameters associated with excitatory and inhibitory outcomes, thereby informing future clinical applications.

**Methods:**

A systematic review was conducted using Web of Science and PubMed, including primary interventional human studies on tFUS and neuromodulation published up to December 2024. Eligible studies comprised single-arm trials, quasi-experimental studies, and randomized controlled trials focusing on excitatory and inhibitory effects and specific ultrasound parameters.

**Results:**

The findings summarize the current evidence on tFUS-induced neuromodulation of motor and sensory functions. Both excitatory and inhibitory effects were observed, particularly in the primary motor cortex, primary somatosensory cortex, thalamus, insula, and dorsal cingulate gyrus. These effects appear to be modifiable through specific stimulation parameters.

**Conclusions:**

tFUS demonstrates both online/ offline excitatory/ inhibitory effects on sensory and motor brain regions under specific stimulation conditions. It holds promise as a potential therapeutic strategy for managing motor and sensory dysfunctions, such as stroke and chronic pain. However, the underlying mechanisms remain incompletely understood, necessitating further investigation.

**Supplementary Information:**

The online version contains supplementary material available at 10.1186/s12984-025-01722-9.

## Introduction

Low-intensity transcranial focused ultrasound (tFUS) has emerged as a novel, non-invasive neuromodulation technique capable of targeting specific brain regions. Unlike other neuromodulation methods, such as transcranial magnetic stimulation (TMS) and transcranial direct current stimulation (tDCS), tFUS delivers mechanical acoustic energy that can penetrate deep brain structures while maintaining high spatial resolution [1–4].

Over the past decades, research has significantly advanced our understanding of the mechanical effects of tFUS on the brain. Numerous studies have explored its role in opening the blood-brain barrier (BBB) when combined with microbubbles, facilitating targeted drug delivery [5–8]. Additionally, tFUS has been investigated for its neuromodulatory capabilities, demonstrating that ultrasound alone can modulate neuronal activity without microbubbles. Both in vitro and in vivo studies have provided valuable insights into the ultrasound-neural tissue interaction, including directly activating neurons [9–12], eliciting motor response [13–15], and modulating cortical excitability [3, 16, 17]. These findings have opened new possibilities for therapeutic applications, particularly in treating neurological [18–20] and psychiatric diseases [21–23].

Despite significant advancements, the specific excitatory and inhibitory impacts on motor and sensory functions, contingent upon varying stimulation parameters, are not fully elucidated. Variations in experimental designs—including outcome measurements, targeted brain regions, and particularly ultrasound parameters (e.g., intensity, frequency, pulse repetition rate, duty cycle, and pulse train duration)—have led to inconsistencies across studies, complicating result interpretation and development of standardized clinical protocols [24–26].

To bridge this gap, a systematic review of tFUS effects is crucial, focusing on its excitatory and inhibitory potentials. Understanding how tFUS influences neural circuits under specific conditions is essential for its safe and effective clinical translation. This review summarizes current evidence on tFUS neuromodulation, particularly its effects on human sensory and motor functions. Additionally, it explores possible clinical applications of tFUS, discussing its therapeutic potential for diseases such as stroke and chronic pain, and challenges for future research.

## Methods

This is a systematic review that adhered to the Preferred Reporting Items for Systematic Reviews and Meta-Analysis (PRISMA) Statement [27]. Web of Science and Pubmed database were queried using search string: *(“transcranial ultrasound” OR “transcranial focused ultrasound” OR “Low intensity focused ultrasound”) AND (“effects” OR “neuromodulation” OR “modulation” OR “modulate” OR “stimulate” OR “stimulation” OR “neuroplasticity” OR “plasticity”) AND (“human” OR “patients” OR “participants” OR “Adults”)*. The retrieved studies were imported into EndNote, where duplicates were removed.

**Fig. 1:**
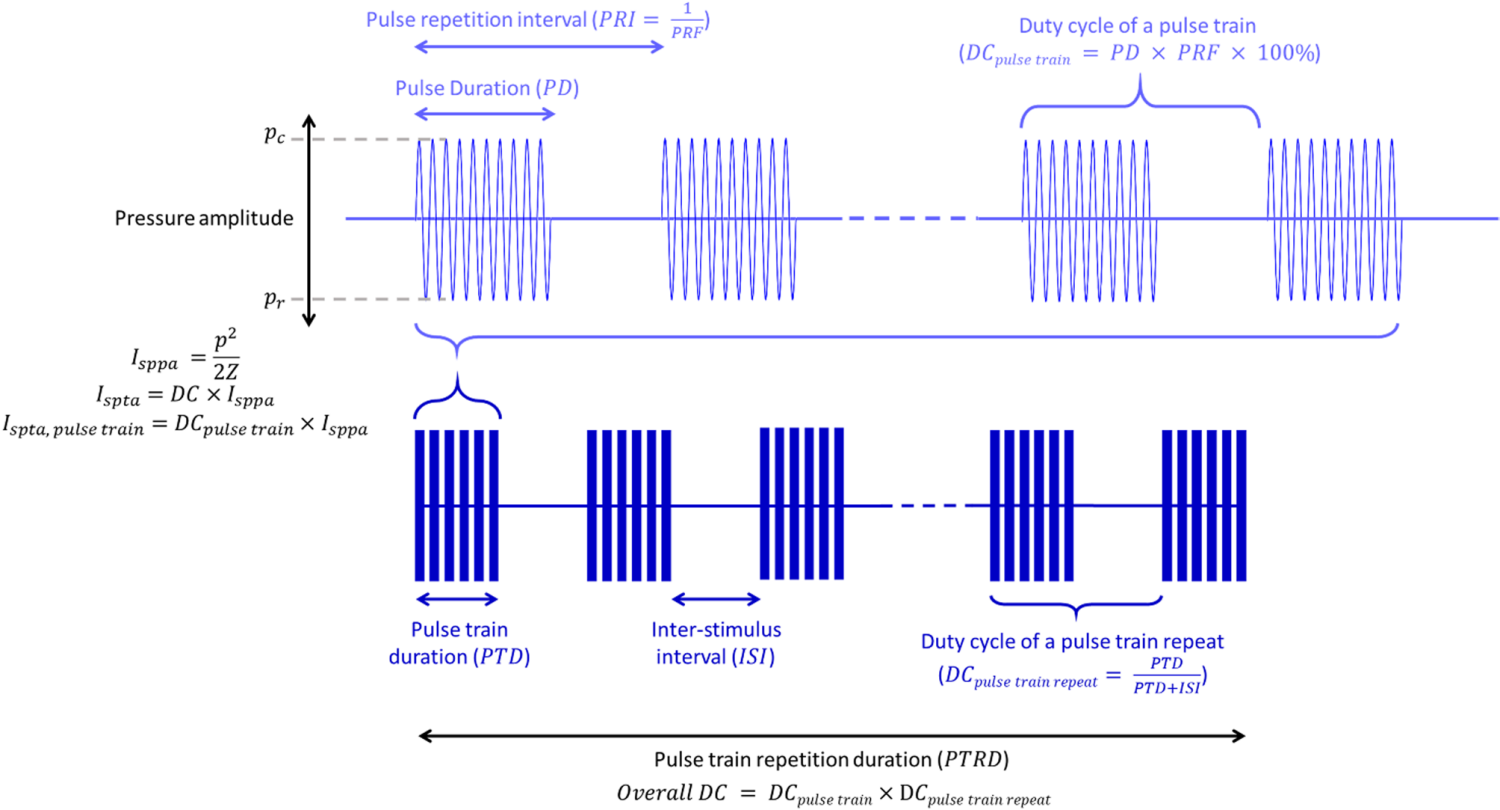
Schematic of ultrasound parameters. Ultrasound stimulation consists of a series of pulses, each repeating at a defined pulse repetition frequency (PRF). The duration of a single pulse is defined as pulse duration (PD). These pulses are delivered in sequences known as pulse trains. The duration of a pulse train is the pulse train duration (PTD), which has been described as “burst duration” in some previous literature. Depending on the experimental protocol, pulse trains may be repeated, and the duration is termed pulse train repetition duration (PTRD). The duty cycle represents the percentage of time that a pulse or pulse train is active. The duty cycle of a pulse train (DC_pulse train_) can be calculated by the product of the PD and PRF, multiplied by 100%. The duty cycle of a pulse train repeat (DC_pulse train repeat_) is defined as the ratio of the PTD to the total stimulation period, including both the PTD and the inter-stimulus interval (ISI). The overall duty cycle (DC) is the product of DC_pulse train_ and DC_pulse train repeat_. The peak pressure is shown as p_c_ and p_r_, from which ultrasound intensity can be derived. (c refers to compression, r refers to rarefaction.) The spatial-peak, pulse-averaged intensity (I_sppa_) indicates the average intensity of a single pulse while the spatial-peak, temporal-averaged intensity (I_spta_) accounts for intensity over the entire experiment. The listed formulas are simplified when there is no ramping and Z denotes the characteristic acoustic impedance of the medium

For inclusion, studies had to utilize transcranial focused ultrasound for neuromodulation on humans from 2014 to 2024. The exclusion criteria are as follows: (1) review articles; (2) studies not conducted on humans; (3) studies focused on ultrasound applications other than brain neuromodulation; (4) studies focused on the physical or material properties of ultrasound; (5) studies that utilized diagnostic ultrasound, non-focused transcranial ultrasound or transcranial pulse stimulation, which has a shorter pulse duration (a few µs) and a different intensity distribution. Abstracts were screened for relevance, and full-text articles were assessed to confirm the studies’ eligibility. For each study, extracted content included bibliographic data, study type, ultrasound targets, ultrasound parameters, sonication protocols, outcome measurements, and a summary of results. The definitions of ultrasound parameters are illustrated in Fig. 1, aligning with ITRUSST consensus [28] and a previous review [29].

## Results

### Literature review

**Fig. 2:**
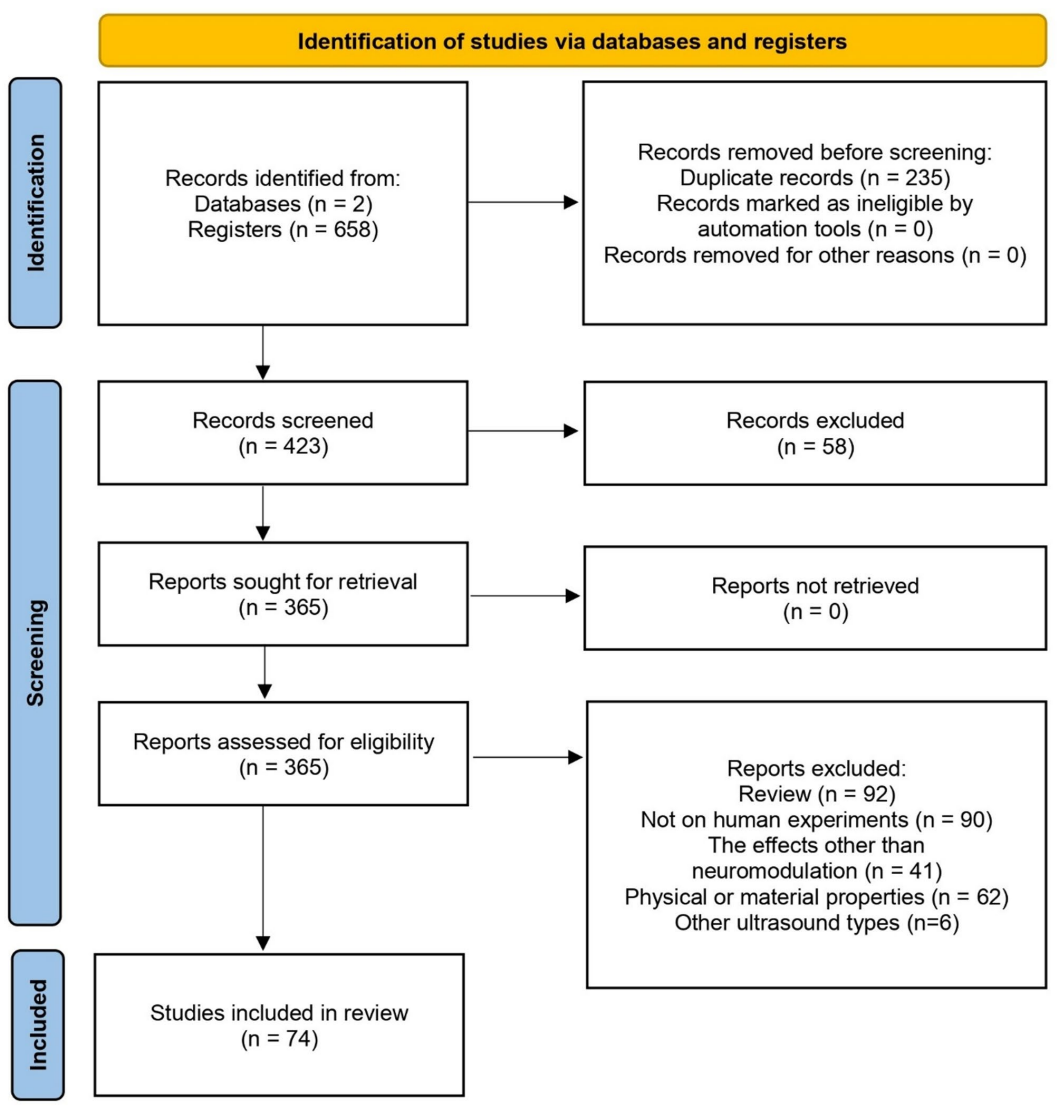
PRISMA 2020 flow diagram. This diagram showed the study selection process

A total of 658 results were initially retrieved. After removing 235 duplicates and excluding 58 irrelevant articles by titles and abstracts, an additional 289 studies were excluded based on the following criteria: (1) 92 studies were review articles; (2) 90 studies were not conducted on humans (e.g., primates, sheep, mice, rats, or stem cell organoids); (3) 41 studies focused on ultrasound applications other than neuromodulation (e.g. ablation and blood-brain barrier opening); (4) 62 studies focused on the physical or material properties of ultrasound; (5) 6 studies utilized diagnostic ultrasound, non-focused transcranial ultrasound or transcranial pulse stimulation. Ultimately, 74 articles were selected for discussion in this review (see PRISMA flow diagram, Fig. 2). Of these, 34 articles focusing on sensory and motor functions form the primary focus of this analysis.

### Motor functions

A total of 21 studies have explored the application of tFUS on motor cortex. Most studies utilized a fundamental frequency ($\:{f}_{0}$) of 0.5 MHz, with a duty cycle of mostly 10 or 30% while the pulse repetition frequency (PRF) ranging from 5 Hz to 1 kHz. Other parameters, both positive findings and negative findings are summarized in Table 1. To provide a clearer analysis, we categorized the main results into online effects (during or immediate after stimulation) and offline effects (lasting beyond stimulation).

**Table 1:** Transcranial focused ultrasound studies on human motor cortex

Author	Study type	f_0_ (MHz)	PRF (Hz)	DC_pulse train_ (%)	PTD (s)	DC_pulse train repeat_ (%)	PTRD (s)	I_SPTA_ (W/cm^2^)	I_SPPA_ (W/cm^2^)	$\:{p}_{r}$(MPa)	MI	Main results	Online/ Offline
Ai, 2016 [30]	Single-arm	0.5–0.86	1000	36–50	0.5	-	-	-	16.95	-	-	No change in BOLD signal.	Online
Ai, 2018 [31]	Randomized, sham-controlled crossover	0.5	1000	36	0.5	8.3	540s (30s on/off)	-	16.95	0.686*	0.97	BOLD signal volume↑, but no differences in BOLD percent signal change.	Online
Legon, 2018 [17]	Randomized, sham-controlled, crossover	0.5	1000	36	0.5	-	-	6.16	17.12	0.636*	0.9	MEPs↓at TMS outputs 90%, 95% and 100%. MEPs↓ at ISI 10-15ms (reflects ICF). Reaction time↓.	Online
Fomenko, 2020 [32]	Randomized, double-blind, crossover	0.5	1000	30	0.5	-	-	2.78	9.26	0.523*	0.74	MEPs↓, no effect on silent period duration, SICI↑, LICI-, SICF-, ICF-, Reaction time↓	Online
			1000	10, 30, 50	0.5			0.93, 2.78, 4.63				DC_pulse train_ of 10% suppressed MEPs significantly.	
			1000	30	0.1–0.5			2.78				PTD of 0.4s and 0.5s suppressed MEPs significantly.	
			200, 500, 1000	30	0.5							MEPs↓, no significant change between different PRF.	
Xia, 2021 [33]	Sham-controlled, crossover	0.5	1000	30	0.5	-	-	2.78	9.26	0.523*	0.74	MEPs↓in ipsilateral cortex.	Online
Yu, 2021 [35]	Randomized, sham-controlled, crossover	0.5	300, 3000	6, 60	0.5	-	-	0.7 (targets)	5.9 (scalp)	-	-	MRCP source profile amplitude ↑	Online
Zhang, 2021 [36]	Randomized, sham-controlled, crossover	0.5	100	5	0.5	5.9	900	0.403	8.053	0.495*	0.70	MEPs↑, stop-signal reaction time↓	Offline
Heimbuch, 2022 [49]	Single-arm	0.5	1000	36	0.3, 0.5	-	-	5.57	15.48	-	-	No change of Cortical Silent Period.	Online
						3–5	818					No significant changes in M1 excitability.	Offline
Nakajima, 2022 [38]	Randomized, double-blind, sham-controlled	0.5	10	30	40	-	-	9	30	0.495*	0.70	MEPs↓ for 60 min	Offline
Samuel, 2022[41]	Randomized, sham-controlled, crossover	0.5	5	10	80	-	-	0.23	2.26		-	MEPs↑, SICI↓, ICF-	Offline
Zeng, 2022 [40]	Randomized, sham-controlled, crossover	0.5	5	10	80	-	-	0.23	2.26		-	MEPs↑, SICI↓, ICF↑	Offline
		0.5	1000	32	0.5	31.3	80	0.72	2.26		-	No significant changes in MEPs.	
Ren, 2023 [37]	Single-blind, sham-controlled	0.5	100	5	0.5	5.9	900	0.4	8.086	0.495*	0.70	MEPs↑in ipsilateral cortex. MEPs↓in contralateral cortex.	Offline
Samuel, 2023 [42]	Randomized, single-blind, sham-controlled, crossover	0.5	5	10	80	-	-	-	2.26	-	-	MEPs↑	Offline
Shamli Oghli, 2023 [43]	Randomized, double-blind, sham-controlled, crossover	0.5	5	10	80	-	-	0.29	2.93	-	-	All drugs significantly reduced tbFUS-induced increases in MEP amplitudes compared to placebo.	Offline
Bao, 2024 [44]	Randomized, sham-controlled, crossover	0.5	5	10	80	-	-	0.24	2.38	0.269*	0.38	MEPs↑	Offline
					2	20	400					No significant changes in M1 excitability.	
Ding, 2024 [45]	Randomized, sham-controlled, crossover	0.5	5	10	80	-	-	0.17	1.69	0.212*	0.3	MEPs↑, SICI-, ICF-, SICF-, LICI- Immediate priming with rTMS increased tbFUS-induced plasticity duration while depotentiation with rTMS eliminate the excitatory effect of tbFUS.	Offline
Ennasr, 2024 [34]	Randomized, crossover	0.5	1000	1, 10, 30, 50, 70	0.1, 0.5	-	-	-	6, 24	-	-	MEPs↓the most at DC_pulse train_ 30%, PTD 0.5s, Isppa 6 W/cm2. And only in this condition, pulse tFUS is more inhibitory than continuous tFUS.	Online
				100	0.07, 0.15	-							
Grippe, 2024 [47]	Case-control, Randomized crossover	0.5	5	10	80	-	-	0.29	2.93	-	-	MEPs↑, SICI-, SICF-	Offline
Stanley Chen, 2024 [48]	Randomized, sham-controlled, crossover	0.5	1000	30	0.5	9.1	160s/set Two sets separated by 5 min	2.81	9.38	0.612	0.87	In subjects having online inhibition, tFUS and concurrent TMS stimulation decreased MEP at post-10 to 30 min.	Offline
Zadeh, 2024 [39]	Randomized, double-blind, sham-controlled	0.25	10, 100, 1000	10	120	-	-	0.5	5	-	-	MEPs↓ in PRF 10 and 100 Hz	Offline
Zeng, 2024 [46]	Randomized, crossover	0.5	2, 5, 10	10	40, 80, 120	-	-	0.23	1.13, 2.26	-	-	MEPs↑, SICI↓, ICF-, SICF↑	Offline
		0.5	5	5, 15	80	-		0.11, 0.34	2.26				

Abbreviation: *f*_0_: fundamental frequency; PRF: pulse repetition frequency; DC_pulse train_: duty cycle of a pulse train; PTD: pulse train duration; DC_pulse train repeat_: duty cycle of a pulse train repeat; PTRD: pulse train repetition duration; I_SPTA_: Intensity Spatial Peak Temporal Average; I_SPPA_: Intensity Spatial Peak Pulse Average; $\:{p}_{r}$: peak-rarefactional pressure; MI: mechanical index; SEPs: somatosensory evoked potentials; MEPs: motor evoked potentials; EEG: electroencephalography; BOLD: blood-level-oxygen dependent; TMS: transcranial magnetic stimulation; rTMS: repetition TMS; MRCP: movement-related cortical potential; M1: primary motor cortex; SICI: short-interval intracortical inhibition; ICF: intracortical facilitation; SICF: short-interval intracortical facilitation; LICI: long-interval intracortical inhibition; tbFUS: theta burst focused ultrasound. $\:{p}_{r}$ can be calculated by the formula: $\:MI=\frac{{p}_{r}}{\sqrt{{f}_{0}}}$ and the calculated values are marked with an asterisk (*). Symbols: ↑: increase; ↓: decrease; -: no change

####  Online effects

tFUS targeting the primary motor cortex (M1) was found to elicit focal blood oxygen level dependent (BOLD) activation in some individuals, as reported by Ai et al. However, no significant group-level changes in BOLD signal were observed, either with a single 0.5 s sonication [30] or with cumulative stimulation lasting 540 s [31]. Subsequent studies have increasingly employed motor-evoked potentials (MEPs) induced by transcranial magnetic stimulation (TMS) as outcome measures to assess the neuromodulatory effects of tFUS.

Legon et al. [17] first conducted a sham-controlled, crossover study and showed that an single burst tFUS decreased MEPs amplitude immediately following sonication with the parameters of: $\:{f}_{0}$: 0.5 MHz, PRF: 1 kHz, DC_pulse train_: 36%, PTD: 0.5s, I_SPPA_: 17.12 W/cm^2^. The results were also reported by subsequent studies using similar parameters [32–34]. Fomenko et al. further systemically examined ultrasound parameters with a blocked design and showed that longer pulse train duration (0.4–0.5 s vs. 0.1–0.3 s) and lower DC_pulse train_ (10% vs. 30–50%) suppressed MEPs more effectively, while PRF of 200 Hz, 500 Hz, and 1000 Hz yielded the same suppression effect [32]. A recent study by Ennasr et al. examined a variety of DC_pulse train_ (1, 10, 30, 50, 70%), PTD (0.1, 0.5 s) and intensity (I_SPPA_: 6, 24 W/cm^2^) at constant 1 kHz PRF and revealed that the greatest inhibition was a combination of DC_pulse train_: 30%, PTD: 0.5 s, and I_SPPA_: 6 W/cm^2^ [34]. Apart from MEP changes, decreased reaction time was also observed during tFUS sonication [17, 32].

Moreover, Xia et al. demonstrated a significant decrease in MEPs amplitude of the ipsilateral M1 at 20 ms after 0.5 s tFUS, without affecting the contralateral M1 [33]. They assumed that tFUS could selectively modulate corticospinal neurons because the callosal projection neurons or interhemispheric connection have higher threshold than corticospinal connections.

The studies showed discrepancy results regarding modulation effect of tFUS on intracortical circuits. Legon et al. [17] showed a significant MEPs decrease at intracortical facilitation (ICF) (inter-pulse interval, IPI: 10–15 ms) and no significant change at short-interval intracortical inhibition (SICI) (IPI: 1–5 ms), with the tFUS beginning 100 ms prior to the conditioning stimulus. On the other hand, Fomenko et al. [32] revealed no significant change of MEPs at ICF but a significant potentiation at SICI with IPI 10ms for ICF and IPI 2ms for SICI, and the tFUS beginning 490ms prior to the first TMS pulse. The inconsistency might result from different IPI selection and the onset timing of tFUS.

tFUS has also been reported to exhibit online excitatory effects when applied with a higher PRF (3000 Hz). With electroencephalogram (EEG) and electrophysiological source imaging (ESI), tFUS ($\:{f}_{0}$: 0.5 MHz, PRF: 3000 Hz, PTD: 0.5 s, I_SPPA_: 5.9 W/cm^2^) significantly increased movement-related cortical potential amplitude from a foot tapping task, indicating an enhancement of cortical activity related to voluntary movement [35].

#### Offline effects

Recent studies have increasingly focused on the offline effects of tFUS, demonstrating that it can induce sustained excitatory or inhibitory outcomes on cortical excitability. Zhang et al. applied tFUS ($\:{f}_{0}$: 0.5 MHz, PRF: 100 Hz, DC_pulse train_: 5%, PTD: 0.5 s, PTRD: 15 min, I_SPPA_: 8.053 W/cm^2^) on M1, resulting in an increase of MEPs amplitude that persisted for 30 min post-stimulation and a decrease of reaction time [36]. Subsequently, a follow-up study with similar parameters and stimulation protocol not only confirmed the excitatory effects of tFUS over the ipsilateral M1 but also reported an inhibitory effect over the contralateral cortex, suggesting a shift in interhemispheric balance [37]. Nevertheless, two double-blind, sham-controlled studies using similar parameters observed a significant decrease in MEPs amplitude, persisting over 60 min after tFUS stimulation [38, 39]. The common characteristics of applied parameters are low PRF (10 and 100 Hz), low DC_pulse train_ (5 and 10%), and longer PTD (40 s and 120 s), but no significant inhibitory or excitatory effects were observed when adjusting PRF to 1000 Hz [39].

It’s worth noting that a new protocol, theta burst focused ultrasound (tbFUS) with PRF of 5 Hz was introduced by Zeng et al. ($\:{f}_{0}$ of 0.5 MHz, PTD of 80 s, DC_pulse train_ of 10%, and I_SPPA_ of 2.26 W/cm^2^) [40]. tbFUS produced a 30-min increase of MEPs amplitude after sonication. Similar excitatory effects of tbFUS were also found by other sham-controlled studies using the same stimulation parameters [41–45]. As for intracortical circuit, the studies showed inconsistent results, with some reporting a decrease in SICI [40, 41, 46], and increase in ICF [40] while some found no change of SICI [43, 45, 47], ICF [41, 43, 45, 46], short-interval intracortical facilitation (SICF) [45, 47] and long-interval intracortical inhibition (LICI) [45]. These studies employed identical TMS stimulation parameters, IPI (2ms for SICI, 10ms for ICF), and MEPs recording time points (before and 5, 30, 60 min after tbFUS). The inconsistency in results may be attributed to individual differences in baseline cortical excitability and the relatively small sample sizes. For example, individuals of older age and those with comorbid Parkinson’s disease might respond differently to tFUS and TMS [47]. Moreover, although two studies observed a trend toward reduced SICI, the findings did not reach statistical significance [43, 45].

Zeng et al. [46] further investigated the effects of different parameters of tbFUS and noted there was no modulation effect on MEP amplitude and intracortical circuit at a lower acoustic intensity (I_SPPA_ of 1.13 W/cm^2^). Furthermore, 5 Hz tbFUS induced a higher and more prolonged MEP amplitude change compared to PRF of 2 Hz and 10 Hz. Finally, higher duty cycles and longer pulse train durations were associated with more prolonged increases of MEPs amplitude. (i.e. DC_pulse train_: 15%>10%>5% and PTD: 120 s > 80 s > 40 s)

Interestingly, a study investigated the interaction between tbFUS and rTMS, reporting that administering rTMS before tbFUS extended the increase in MEP amplitudes from 30 to 90 min, while applying rTMS after tbFUS abolished this plasticity [45]. A recent study also demonstrated the interactive effect of tFUS and single-pulse TMS. In subjects having online inhibition, two blocks of 30 times of 0.5s tFUS and concurrent TMS stimulation decreased MEP at 10 to 30 min post-stimulation [48]. The offline inhibitory effect was not observed when applying tFUS and TMS separately stimulation, or applying TMS alone, and tFUS did not have inhibitory effect in the subjects failing to have online inhibition, indicating a potential interplay between these two neuromodulation techniques.

With regard to mechanisms, a randomized, double-blind, placebo-controlled pharmacological study proved that the excitatory effects of tbFUS were related to sodium channels, calcium channels, as well as GABA_A_ and NMDA receptors [43]. These findings support the hypothesis that tbFUS induces long-term potentiation (LTP)-like mechanisms, likely mediated by the activation of mechanosensitive sodium and calcium channels.

The first clinical application of tFUS on motor cortex was reported by Samuel et al. [42], who applied tbFUS ($\:{f}_{0}$: 0.5 MHz, PRF: 5 Hz, DC_pulse train_: 10%, PTD: 80 s, and I_SPPA_: 2.26 W/cm^2^) to people with Parkinson’s disease (PD), exhibiting an increase of MEPs up to 30 min, compared to the sham condition, but no significant changes were observed in MDS-UPDRS-III scores. Grippe et al. further found that the increase of MEPs and improved bradykinesia scores only occurred in medicated PD people but not in those off medication [47]. The results indicate tFUS as a potential non-invasive treatment option to complement existing PD therapies.

It should be noted that there are some study designs and parameters showing no significant effects of tFUS (see Table 1 for specific parameters). The reasons for this inconsistency are unclear, but several observations may offer some insight. Firstly, tFUS did not produce significant change on cortical silent period [32, 49], which is believed to be a marker of GABA_B_ activity. This indicates that the effect of tFUS may not be mediated through GABA_B_ mechanism. Secondly, ISI seemed to be a critical parameter for offline effects. For example, Zeng et al. [40] and Bao et al. [44] utilized intermittent tFUS with ISIs of 1.1s and 8s respectively and didn’t observe significant MEPs change, while continuous tFUS increased MEPs after stimulation [40, 44]. Notably, that excitatory offline effects have also been reported with tFUS using ISI, warranting further investigation into how parameters affect the interplay between tFUS and neurons.

**Fig. 3:**
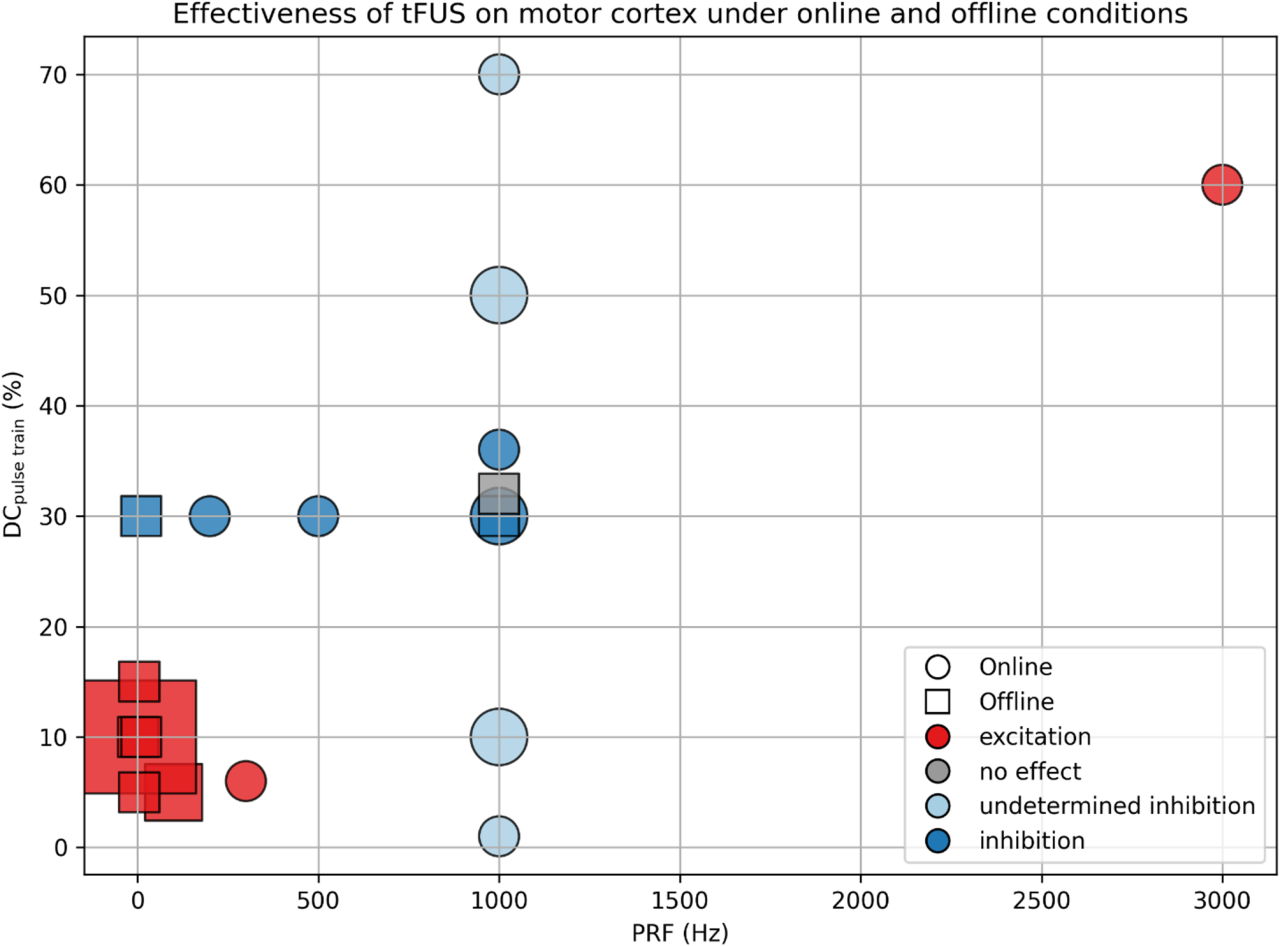
Effectiveness of tFUS on motor cortex under online and offline conditions

As far as the above articles are concerned, we summarize the parameters associated with the most consistent results in Supplementary Table 1. Additionally, we selected high-quality studies (i.e., randomized controlled trials) that employed the most commonly used $\:{f}_{0}$ of 0.5 MHz, and visualized their DC_pulse train_ and PRF in a scatter plot (Fig. 3). Two trends are particularly robust, supported by validation from multiple studies. Firstly, tFUS induced an online inhibitory effect (decreased MEPs amplitude and increased SICI during tFUS) with $\:{f}_{0}$ of 0.5 MHz, a PRF of 1 kHz, a DC_pulse train_ of 30–36%, and a PTD of 0.4–0.5 s. Second, tFUS induced an offline excitatory effect (increased MEPs amplitude and decreased SICI after tFUS, lasting 5–30 min) with $\:{f}_{0}$ of 0.5 MHz, a PRF of 5 Hz, a DC_pulse train_ of 10%, and a PTD of 80–120 s.

Each marker represents a study condition, plotted according to pulse repetition frequency (PRF, x-axis) and duty cycle (DC_pulse train_, y-axis). Marker shapes indicate the effects timing (circles: online; squares: offline), while marker colors indicate observed outcomes (e.g., excitation, inhibition, no effect). “Undetermined inhibition” refers to conditions with inconsistent findings across studies. Marker size reflects the number of studies per condition. The results suggest that tFUS induce an online inhibitory effect with a PRF of 1 kHz and a DC_pulse train_ of 30–36%, and an offline excitatory effect with a PRF of 5 Hz and a DC_pulse train_ of 10%. However, tFUS induce inconsistent online MEPs change with a PRF of 1 kHz and DC_pulse train_ of 1, 10, 50, 70%, and having no offline MEPs change with a PRF of 1 kHz, a DC_pulse train_ of 32% and an ISI of 1.1 s (ISI is not shown in this figure). References: [17, 32, 34–38, 41–48, 50].

### Sensory functions

A total of 13 literatures have examined tFUS effects on human sensory functions. The target brain regions included the primary somatosensory cortex (S1), secondary somatosensory cortex (S2), thalamus, insula, and cingulate cortex. All the positive and negative findings are summarized in Table 2. Most of the studies focused on the online effects.

**Table 2:** Transcranial focused ultrasound studies on human sensory functions

Author	Study type	Target	f_0_ (MHz)	PRF (Hz)	DC_pulse train_ (%)	PTD (s)	DC_pulse train repeat_ (%)	PTRD (s)	I_SPTA_ (W/cm^2^)	I_SPPA_ (W/cm^2^)	$\:{p}_{r}$(MPa)	MI	Main results	Online/ Offline
Legon, 2014 [3]	Randomized, sham-controlled, crossover	S1	0.5	1000	36	0.5	-	-	-	23.87	0.8	1.13	SEPs↓, Sensory discrimination↑.	Online
Muller, 2014 [4]	Randomized, sham-controlled, crossover	S1	0.5	1000	36	0.5	-	-	-	23.87	0.8	1.13	Alter EEG oscillatory dynamics.	Online
Lee, 2015 [52]	Randomized, sham-controlled, crossover	S1	0.25	500	50	0.3	-	-	1.5	3	0.31*	0.62	Elicited sensory sensations with an evoked-potential.	Online
Lee, 2016 [54]	Randomized, sham-controlled, crossover	S1, S2	0.21	500	50	0.5	-	-	17.5	35	-	-	Elicited various tactile sensations.	Online
Legon, 2018 [55]	Randomized, sham-controlled, crossover	VPL	0.5	1000	36	0.5	-	-	-	14.56	0.63*	0.89	SEPs↓, tactile discrimination task↓.	Online
Badran, 2020 [56]	Randomized, double-blind, sham-controlled, crossover	Anterior thalamus	0.65	10	5	30	50	1200	0.995	14.38	0.72	0.89	Thermal pain threshold↑.	Offline
Liu, 2021 [51]	Randomized, sham-controlled, crossover	S1	0.5	300	6	0.5	-	-	0.34	5.64	0.78	1.10*	S1 activity↑, Sensory discrimination↑	Online
Kim, 2023 [53]	Randomized, sham-controlled, crossover	S1 & VPL	0.25	1400, 700, 350	70	0.2	-	-	-	14.7 W/cm2 for S1, 9.1 W/cm2 for VPL	-	-	Elicited distinctive evoked potential, similar to SEPs, PRF 1400 Hz was more distinct than other PRFs.	Online
							4.8	1280					Increased functional connectivity in precentral gyrus, insula, and supplementary motor area.	Offline
In, 2024 [60]	Randomized, double-blind, sham-controlled, crossover	insula	0.5	1000	36	1	16.7	600	1.5	4.2	0.38	0.2	tFUS did not affect pain ratings during the CPM tasks. tFUS to the PI reduces temporal summation of pain.	Offline
		dACC							1.62	4.5	0.40	0.23		
Legon, 2024 [58]	Double-blind, sham-controlled, crossover	insula	0.5	1000	36	1	-	-	5.38	3.5	0.4	0.57*	tFUS to AI & PI: reduced pain-evoked potential amplitudes. tFUS to PI: reduced pain rating tFUS to AI: reduced EEG power and increased metrics of heart-rate variability.	Online
Jang, 2024 [57]	Pseudo-randomized, crossover	thalamus	0.65	10	70, 5	30	50	720	0.72	-	-	-	Sensitivity↑ when targeting VA thalamus with DC_pulse train_ of 70%. Categorization accuracy↓ when targeting all thalamic regions with DC_pulse train_ of 70%. Decision bias↑when targeting all thalamic regions with DC_pulse train_ of 5%.	Online
Strohman, 2024 [61]	Randomized, double-blind, sham-controlled, crossover	insula	0.5	1000	36	1	16.7	600	1.5	4.2	0.38	0.2	tFUS to the PI decreased HEP amplitudes. tFUS did not affect HRV with no pain stimuli.	Online
		dACC							1.62	4.5	0.40	0.23		
Strohman, 2024 [59]	Randomized, double-blind, sham-controlled, crossover	dACC	0.5	1000	36	1	-	-	1.56	4.34	0.37	0.52*	Pain ratings↓ HRV↑	Online

Abbreviation: S1: primary somatosensory cortex; S2: secondary somatosensory cortex; SEP: sensory-evoked potential; VPL: ventral posterolateral nucleus; CPM: Conditioned Pain Modulation; PI: posterior insula; AI: anterior insula; dACC: dorsal anterior cingulate cortex; VA thalamus: ventral anterior nucleus of thalamus; HRV: heart-rate variability; HEP: heartbeat-evoked potential. Either $\:{p}_{r}$ or $\:MI$ can be calculated from the other using the formula: $\:MI=\frac{{p}_{r}}{\sqrt{{f}_{0}}}$ and the calculated values are marked with an asterisk (*). Symbols: ↑: increase; ↓: decrease

#### Sensory modulation on somatosensory cortex

Accumulating studies have demonstrated that tFUS targeting S1 can modulate sensory functions. These outcomes can be broadly categorized into mechanistic, intermediate, and clinically functional effects.

##### Mechanistic outcomes

Focus on underlying neural dynamics. With the parameters of 0.5 MHz frequency, 1000 Hz PRF, 36% DC_pulse train_, 23.87 W/cm^2^ I_SPPA_, and 0.5s PTD, tFUS reduced the EEG power of alpha-, beta-, and gamma-band activity in specific time windows [3], and modulated phase rate distribution [4]. These effects may result from local recurrent inhibitory mechanisms that help maintain the excitation-inhibition balance.

##### Intermediate outcomes

Such as changes in sensory-evoked potentials (SEPs), further support tFUS-induced neural modulation. Legon et al. demonstrated that tFUS significantly reduced the amplitude of both short-latency and long latency SEPs, elicited by median nerve (MN) stimulation [3]. In addition, tFUS with a lower PRF (300 Hz), a lower DC_pulse train_ (6%), and a lower I_SPPA_ (5.64 W/cm^2^) significantly increased sensory source profile amplitude in the EEG source domain [51]. Furthermore, tFUS has been shown to induce potential changes. Two studies applied tFUS to S1 using different parameters, yet both produced evoked-EEG potentials resembling SEPs elicited by MN stimulation [52, 53].

##### Clinically functional outcomes

Those with direct behavioral relevance—have also been observed. tFUS to S1 improved performance in tasks such as two-point discrimination [3] and frequency discrimination [3, 51]. Lee et al. reported an overall 54.4% sensations rate with tFUS stimulation ($\:{f}_{0}$ 0.25 MHz, PRF 500 Hz, DC_pulse train_ 50%, PTD 0.3 s, and I_SPPA_ 3 W/cm^2^) [52], while Kim et al. elicited tactile sensations in 3 out of 8 participants using a higher PRF (1400 Hz), higher DC_pulse train_ (70%), and higher I_SPPA_ (14.7 W/cm^2^) [53]. Another study by Lee et al., tFUS on S1 achieved a higher sensation rate to 68%, with a lower frequency (0.21 MHz), a longer PTD (500 ms), and an even higher I_SPPA_ (35 W/cm^2^) [54]. In addition, simultaneous stimulation on S1 and S2 elicited various tactile sensations with a 61% response rate [54].

#### Sensory modulation on thalamus

##### Intermediate outcomes

Kim et al. demonstrated that tFUS with 1400 Hz PRF and 70% DC_pulse train_ targeting the ventral posterior lateral (VPL) nucleus increased functional connectivity between the sonicated VPL and regions such as the precentral gyrus, insula, and supplementary motor area, indicating that tFUS may modulate network activity associated with the target regions [53]. In addition, tFUS at the VPL yielded distinct tFUS-mediated evoked potentials [53]. Legon’s group has demonstrated that tFUS targeting the VPL nucleus decreased SEPs during stimulation [55].

##### Clinically functional outcomes

Contrary to the results reported with tFUS at S1, tFUS at VPL reduced tactile discrimination ability [55]. A double-blind, randomized controlled study targeting the right anterior thalamus also demonstrated a significant increase in thermal pain threshold without changes in sensory or tolerance thresholds [56]. Recently, Jang et al. investigated the distinct roles of thalamic regions in conscious perception and reported that tFUS applied to the ventral anterior thalamus enhanced visual perception sensitivity with a 70% DC_pulse train_ but not a 5% DC_pulse train_. Additionally, tFUS applied to all thalamic regions (i.e. ventral anterior, ventral posterior, dorsal anterior, and dorsal posterior nuclei) decreased categorization accuracy at a 70% DC_pulse train_ while increased decision bias at a 5% DC_pulse train_ [57]. It should be noted that this article was still a preprint until the submission.

These results suggest that tFUS targeting different thalamic regions can influence functional connectivity in sensation and possibly decrease sensory and pain perception.

#### Neural circuits modulation on insula and dorsal anterior cingulate cortex

Four double-blind, sham-controlled, crossover studies explored the tFUS effects on the insula and dorsal anterior cingulate cortex (dACC) using the same parameters ($\:{f}_{0}$: 0.5 MHz, PRF: 1000 Hz, DC_pulse train_: 36%, PTD: 1s) but with different outcome measures. Regarding pain perception, tFUS applied to the posterior insula (PI) and dACC, but not the anterior insula (AI) reduced thermal pain perception [58–60]. Additionally, both the AI and PI stimulation reduced pain-evoked potential amplitudes [58]. In terms of autonomic regulation, tFUS to both the AI and dACC increased heart rate variability (HRV) during heat stimulus, such as standard deviation of the RR interval and low frequency power [58, 59], indicating enhanced autonomic stability during pain. In addition, tFUS targeting the PI decreased heartbeat-evoked potential (HEP) [61], emphasizing the role of the PI in interoceptive signal modulation by receiving and integrating direct cardiac input via the thalamus. However, in the absence of a painful stimulus, tFUS to the AI, PI, or dACC did not significantly alter HRV [61], supporting the idea that tFUS effects may be state-dependent.

In summary, these studies suggest that tFUS can exert both inhibitory and excitatory effects on distinct brain regions including the somatosensory cortex and thalamus, and possibly influence neural circuits related to somatosensory processing and pain perception, highlighting its potential for pain management.

**Fig. 4:**
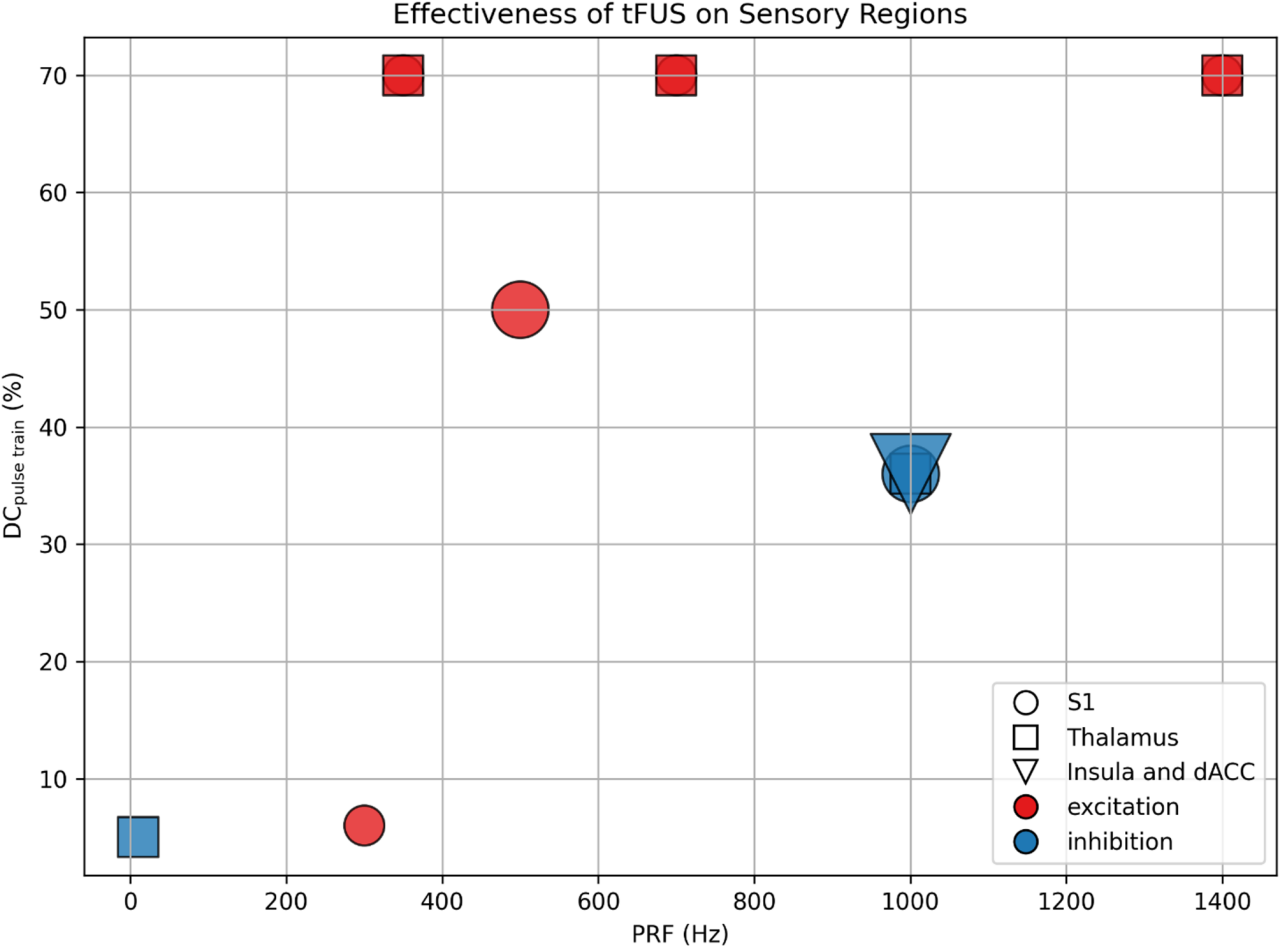
Effectiveness of tFUS on sensory regions

As reviewed in the above studies, the distinct parameters associated with the most consistent excitatory/inhibitory effects are summarized in Supplementary Table 2. We further selected published high-quality studies (i.e., randomized controlled trials) and visualized their DC_pulse train_ and PRF in a scatter plot (Fig. 4). Two trends appear to be readily evident. tFUS exerts inhibitory effects on sensory cortex, thalamus, insula, and dACC with $\:{f}_{0}$ of 0.5 MHz, a PRF of 1000 Hz, a DC_pulse train_ of 36%, and PTD of 0.5–1 s. In contrast, tFUS tends to have excitatory effect on sensory cortex and thalamus with a higher DC_pulse train_ (50–70%).

Each marker represents a study condition, plotted according to pulse repetition frequency (PRF, x-axis) and duty cycle (DC_pulse train_, y-axis). Marker shapes indicate the target regions (circles: S1; squares: thalamus; triangle: insula and dACC), while marker colors indicate observed outcomes (e.g., excitation, inhibition). Marker size reflects the number of studies per condition. The results suggest that tFUS exerts inhibitory effects on sensory cortex, thalamus, insula, and dACC with a PRF of 1000 Hz and a DC_pulse train_ of 36%. In contrast, tFUS produce excitatory effects on sensory cortex and thalamus with a higher DC_pulse train_ (50–70%). References: [3, 4, 51–56, 58–61].

### Adverse effect

A total of 690 participants received tFUS stimulation across the included 34 studies. Among them, four studies reported long-term follow-up outcomes (ranging from several days to months), while the remaining studies assessed only short-term effects (within the day of stimulation). No major adverse events were reported in any study. Eight studies documented mild to moderate transient symptoms experienced by participants during and/or after tFUS stimulation, as summarized in Supplementary Table 3. All reported symptoms had an incidence of less than 10%, with the most common being scalp sensations (9.01%), sleepiness (7.17%), and neck pain (5.93%). All symptoms resolved spontaneously without any persistent discomfort. These findings support the conclusion that under the present conditions, tFUS is a safe neuromodulation technique for modulating motor and sensory function.

## Discussion

In this review, we summarized the current tFUS neuromodulation effects on motor and sensory functions. Both inhibitory and excitatory effects have been observed, particularly in the modulation of the primary motor cortex, the primary somatosensory cortex, the thalamus, the insula, and the dorsal cingulate gyrus, with the nature of these effects potentially adjustable through specific stimulation parameters. In addition, there is a total of 19 studies targeting other brain regions, the parameters and main outcomes are summarized in Supplementary Table 4 [38, 62–79].

Based on these results, we hypothesized that tFUS could emerge as a novel treatment strategy for stroke. In people with motor cortex stroke, impaired motor function is associated with decreased MEPs and attenuated SICI [80]. Besides, the contralateral M1 becomes overactivated during movement execution of the affected limb [81]. An interhemispheric imbalance model suggested that hemiparesis results not only from damaged corticospinal output from the affected hemisphere but also from excessive inhibition from the contralesional hemisphere. As motor function recovers, MEP amplitudes tend to increase [82–84], and people with stroke gradually restore interhemispheric balance across the bilateral sensorimotor cortices and premotor cortex [85]. This model has served as the foundation for neuromodulation techniques, such as repetitive transcranial magnetic stimulation (rTMS), intermittent theta-burst stimulation (TBS) and transcranial direct current stimulation (tDCS) to reduce excitability in the unaffected hemisphere and enhance the cortical excitability in the affected hemisphere [86, 87]. Given its potential for parameter-specific modulation, tFUS may serve as a promising treatment for stroke, with inhibitory stimulation applied to the contralateral M1 and excitatory stimulation targeting ipsilateral M1. A recently published phase 1 trial applied tFUS ($\:{f}_{0}$: 0.5 MHz, PRF: 1 kHz, DC_pulse train_: 20%, PTD: 0.5s, I_SPPA_: 0–8 W/cm^2^) to the contralateral M1 in 18 individuals with stroke. No adverse events were reported among participants. The study reported a significantly higher percentage of patients in the high-intensity group (I_SPPA_: 4–8 W/cm²) showed improvement in motor tasks and greater motor-evoked potentials compared to the low-intensity group (I_SPPA_: 0–2 W/cm²) [88]. Although the definite neuromodulatory effects of tFUS remain to be elucidated, this study supports our hypothesis that tFUS may serve as a potential treatment for stroke. Nonetheless, further translational research is essential to validate these hypotheses.

The other possible application is chronic pain, which an estimated 20.4% of people suffer from and is associated with psychiatric disease and reduced quality of life [89]. Many brain regions participate in the complicated pathophysiology, for example, the motor cortex, sensory cortex, thalamus, insula, anterior cingulate cortex [90]. Multiple non-invasive neuromodulation modalities have been utilized to supplement the treatment. A meta-analysis showed that rTMS exerted some benefits for neuropathic pain and headaches and tDCS significantly lowered pain intensity and had positive effects for quality of life [91]. Recently, tFUS targeting S1 and the insula significantly suppressed pain in a chronic pain mouse model [92]. Furthermore, a double-blind, randomized, controlled trial demonstrated that tFUS to the anterior cingulate cortex significantly reduced pain ratings in people with chronic pain [93]. Based on the findings of this review, inhibitory tFUS stimulation of S1, M1, insula, thalamus, and anterior cingulate cortex has the potential to play a role in the treatment of chronic pain. With its ability to achieve deep penetration and high spatial resolution, tFUS offers a non-invasive approach to precisely target pain-related nuclei, enhancing its therapeutic viability.

To provide a clearer framework for future studies, we summarized proposed applications of tFUS across selected neurological conditions in Table 3. This table maps each condition to its hypothesized brain targets, intended modulation goals, and supporting tFUS parameters derived from the concluded human studies.

**Table 3:** Proposed guiding framework for tFUS applications in neurological disorders

Neurological conditions	Proposed brain targets	Modulation goal	Supporting tFUS parameters
Stroke	Contralateral motor cortex	Inhibition	$\:{f}_{0}$: 0.5 MHz, PRF: 1 kHz, DC_pulse train_: 30–36%, PTD: 0.4–0.5 s.
	Ipsilateral motor cortex	Excitation	$\:{f}_{0}$: 0.5 MHz, PRF: 5 Hz, DC_pulse train_: 10%, PTD: 80–120 s.
Chronic pain	Sensory cortex, thalamus, insula and dACC	Inhibition	$\:{f}_{0}$: 0.5 MHz, PRF: 1000 Hz, DC_pulse train_: 36%, PTD: 0.5–1 s.

It’s worth noting that transcranial pulse stimulation (TPS), a specific type of acoustic pulses, has been supported by several studies demonstrating its safety and effectiveness in individuals with Alzheimer’s disease [94–96], dementia [97, 98], depression [99], autism spectrum disorder [100], and Parkinson’s disease [101, 102]. As for motor and sensory function, TPS enhanced functional brain network when targeting to S1 [103], and improved tremor when targeting to contralateral M1 in Parkinson’s disease [102]. However, in this review, we chose to exclude TPS-related studies due to fundamental differences in the mechanisms and stimulation parameters between tFUS and TPS. First, TPS is characterized by single ultrashort pulses (3 µs), repeated every 200–300 ms, while tFUS delivers long trains of pulses. Notably, TPS typically exhibits a broad, multifrequency bandwidth, whereas tFUS operates within a narrower frequency range optimized for various purposes. Furthermore, TPS generates substantially higher negative pressure than tFUS (approximately 6–8, up to 25 MPa vs. ~1 MPa), but typically lower I_spta_. Given our primary objective to identify specific stimulation parameters associated with excitatory and inhibitory outcome, we are concerned that including TPS studies could confound the interpretation of the results due to the distinct stimulation profiles. Future investigations directly comparing TPS and tFUS under matched conditions will be essential to fully elucidate the unique and potentially overlapping mechanisms of these techniques.

## Challenges and future implications

Currently, several challenges remain. Firstly, the underlying mechanisms remain incompletely understood. Two theoretical models have been proposed to elucidate these phenomena: the neuronal intramembrane cavitation excitation (NICE) model and mechanosensitive ion channels. NICE model was proposed by Plaksin, Shoham and Kimmel [104–106]. It suggests that stable cavitation plays a role in action potential generation during ultrasound stimulation. The alternating pressures produced by ultrasound cause the cell membrane bilayer to expand and compress, with nanobubbles periodically contracting and expanding, modulating membrane curvature and neuronal excitability. Moreover, NICE model suggests that ultrasound can selectively activate different cortical neuron subtypes based on proper selection of ultrasound targets and parameters.

Accumulating evidence indicates the vital role of mechanosensitive ion channels in tFUS mediated neuromodulation. These include channels of the two-pore domain (K2P) potassium channel family [107–109], piezo channels [110, 111], and the transient receptor potential (TRP) families [112]. One probable mechanism is that ultrasound directly alters the conformation of these proteins, affecting their permeability, ion influx, and thereby modulating neuronal excitability. Additionally, ultrasound-induced changes in membrane potential could indirectly activate some voltage-gated ion channels, providing another possible explanation for its effects [107, 112–114].

Secondly, the optimal stimulation parameters require further investigation. Studies have demonstrated that various parameters, such as intensity, pulse repetition frequency, duty cycle, and pulse train duration significantly influence the outcomes of neuromodulation. As indicated by the results of this review, both excitatory and inhibitory effects were observed following tFUS, depending on these parameters and targeted brain regions. Understanding the interplay of these factors is essential for determining the precise parameter combinations that maximize the therapeutic potential of tFUS while minimizing variability and unintended side effects.

Thirdly, several studies have acknowledged the impact of individual variability [36, 37, 52, 54]. The differences in skull character, brain structure, and baseline cortical excitability may affect the precision and efficacy of acoustic energy delivery [115]. In general, thicker and denser skulls lead to greater acoustic attenuation [116, 117]. The excessive variations of skull shape, thickness, bony density and structural heterogeneity significantly affected the acoustic energy distribution, complicating accurate focusing on the target regions [117–119]. Furthermore, there is currently a lack of reliable physiological measures to assess the effects of tFUS. The majority of outcome measures relies on MEPs and SEPs, which can vary substantially within and between participants. These results together hurdle the efficacy and the reproducibility of tFUS studies.

Several approaches have been developed to improve targeting accuracy and penetration efficacy. Focused ultrasound, in contrast to planar ultrasound stimulation, minimizes reflections and pressure losses during propagation through the skull. Personalized acoustic simulations based on individual CT scans—used to assess skull density and anatomical structure—further improve targeting accuracy [119]. Moreover, compared to single-element transducers, phased array systems enable dynamic modulation of the focal point by individually adjusting each transducer element [120, 121]. Such individualized modeling significantly enhances the precision and efficacy of ultrasound-based neuromodulation.

Additionally, the potential influence of auditory confounds remains unclear. Several studies have demonstrated that tFUS produce auditory activation, and auditory masking eliminate its effects [122–124], highlighting the need to consider auditory confounds in future research. Although some studies have incorporated auditory masking [32, 40, 48, 58–61], further research is required to definitively distinguish the direct neuromodulatory effects of tFUS from auditory-related influences.

Despite the challenges, tFUS is a promising therapeutic tool for various neurological and psychiatric diseases. Its ability to non-invasively target both superficial and deep brain regions with high precision offers a novel approach to neuromodulation, which may be particularly beneficial for conditions such as stroke and chronic pain. Studies have explored its effects in conditions like consciousness disorder [18, 125], chronic pain [93], Parkinson’s disease [19, 42, 47], Alzheimer disease [19, 126, 127], epilepsy [121, 128, 129], depression [23, 130, 131], anxiety [21], substance use disorder [22, 132], and essential tremor [20, 133, 134]. A thorough review regarding focused and non-focused transcranial ultrasound have been completed by Matt et al. [135]. Future research should prioritize refining theoretical models, conducting rigorous clinical trials with auditory confounds, and exploring long-term safety and efficacy to solidify its roles in both basic neuroscience and therapeutic applications.

## Conclusion

In conclusion, tFUS has demonstrated both excitatory and inhibitory neuromodulatory effects on motor and sensory brain regions. With possible parameter-dependent effects on neurons, we highlight its potential as a versatile neuromodulation tool for neuroscience research and clinical applications, such as stroke, chronic pain and other motor and sensory dysfunctions.

## Supplementary Information

Below is the link to the electronic supplementary material.

Supplementary Material 1

Supplementary Material 2

Supplementary Material 3

Supplementary Material 4

## Data Availability

All data generated or analysed during this study are included in this published article.

## Abbreviations

**Stimulation modality: **tFUS: Transcranial focused ultrasound
tbFUS: Theta burst focused ultrasound
TMS: Transcranial magnetic stimulation
rTMS: Repetitive TMS
tDCS: Transcranial direct current stimulation
TBS: Theta-burst stimulation
**Ultrasound parameters: **f_0_: Fundamental frequency
PRF: Pulse repetition frequency
DC_pulse train_: Duty cycle of a pulse train
PTD: Pulse train duration
PTRD: Pulse train repetition duration
ISI: Interstimulus interval
DC_pulse train repeat_: Duty cycle of a pulse train repeat
I_SPTA_: Intensity spatial peak temporal average
I_SPPA_: Intensity spatial peak pulse average
$\:{p}_{c}$: Peak-compression pressure
$\:{p}_{r}$: Peak-rarefactional pressure
MI: Mechanical index
**Brain regions: **M1: Primary motor cortex
S1: Primary somatosensory cortex
S2: Secondary somatosensory cortex
VPL: Ventral posterolateral nucleus
PI: Posterior insula
AI: Anterior insula
dACC: Dorsal anterior cingulate cortex
VA thalamus: Ventral anterior nucleus of thalamus
STN: Subthalamic nucleus
PFC: Prefrontal cortex
GP: Globus pallidus
rIFG: Right inferior frontal gyrus
DMN: Default mode network
aIFC: Anterior inferior frontal cortex
ErC: Entorhinal cortex
PCC: Posterior cingulate cortex
VC/VS: Ventral capsule/ventral striatum
NAc: Nucleus accumbens
**Outcome measures: **SEPs: Somatosensory evoked potentials
MEPs: Motor evoked potentials
EEG: Electroencephalography
ESI: Electrophysiological source imaging
BOLD: Blood-level-oxygen dependent
MRCP: Movement-related cortical potential
IPI: Inter-pulse interval
SICI: short-interval intracortical inhibition
ICF: Intracortical facilitation
SICF: Short-interval intracortical facilitation
LICI: Long-interval intracortical inhibition
MFT: Midfrontal theta activity
CPM: Conditioned pain modulation
HRV: Heart-rate variability
HEP: Heartbeat-evoked potential
VEP: Visual evoked potential
EMG: Electromyography
rsFC: Resting-state functional connectivity
**Others: **PD: Parkinson’s disease
LTP: Long-term potentiation
MN: Median nerve
NICE model: The neuronal intramembrane cavitation excitation model
K2P: Two-pore domain
TRP: Transient receptor potential
