# Supplementary material for: Neuromodulation effects of low-intensity transcranial focused ultrasound in human, a systematic review focusing on motor and sensory functions
Source: J Neuroeng Rehabil. 2025 Nov 28;22:254. doi: 10.1186/s12984-025-01722-9 (PMC12664190; doi:10.1186/s12984-025-01722-9)
Supplement: Supplementary file 1 — Supplementary Material 1 [file 12984_2025_1722_MOESM1_ESM.docx]

**Supplementary Table 1. The tFUS parameters and the main effects on motor cortex**

| Targets | $f_{0}$ (MHz) | PRF  (Hz) | DC_pulse train_ (%) | PTD  (s) | I_SPTA_ (W/cm^2^) | I_SPPA_  (W/cm^2^) | $p_{r}$(MPa) | MI | Main  effects | Online/  Offline |
| --- | --- | --- | --- | --- | --- | --- | --- | --- | --- | --- |
| Motor cortex | 0.5 | 1000 | 10-36 | 0.4-0.5 | 0.93-6.16 | 6-17.12 | 0.52-0.64 | 0.74-0.90 | Inhibition | Online |
|  | 0.5 | 300, 3000 | 6, 60 | 0.5 | 0.7 | 5.9 | - | - | Excitation | Online |
|  | 0.5 | 10 | 30 | 40 | 9 | 30 | 0.495 | 0.70 | Inhibition | Offline |
|  | 0.25 | 10, 100 | 10 | 120 | 0.5 | 5 | - | - |  |  |
|  | 0.5 | 5 | 10-15 | 80-120 | 0.17-0.34 | 1.69-2.93 | 0.21-0.27 | 0.3-0.38 | Excitation | Offline |

Abbreviation: $f_{0}$: fundamental frequency; PRF: pulse repetition frequency; DC_pulse train_: duty cycle of a pulse train; PTD: pulse train duration; I_SPTA_: Intensity Spatial Peak Temporal Average; I_SPPA_: Intensity Spatial Peak Pulse Average; $p_{r}$: peak-rarefactional pressure; MI: mechanical index
