# Supplementary material for: Neuromodulation effects of low-intensity transcranial focused ultrasound in human, a systematic review focusing on motor and sensory functions
Source: J Neuroeng Rehabil. 2025 Nov 28;22:254. doi: 10.1186/s12984-025-01722-9 (PMC12664190; doi:10.1186/s12984-025-01722-9)
Supplement: Supplementary file 2 — Supplementary Material 2 [file 12984_2025_1722_MOESM2_ESM.docx]

**Supplementary Table 2. The tFUS parameters and the main effects on sensory functions**

| Targets | $f_{0}$ (MHz) | PRF  (Hz) | DC_pulse train_ (%) | PTD  (s) | DC_pulse train repeat_ (%) | PTRD (s) | I_SPTA_ (W/cm^2^) | I_SPPA_ (W/cm^2^) | MI | Main effects | Online/  Offline |
| --- | --- | --- | --- | --- | --- | --- | --- | --- | --- | --- | --- |
| S1 & VPL | 0.5 | 1000 | 36 | 0.5 |  | - | - | 14.56-23.87 | 0.89-1.13 | Inhibition | Online |
| Insula & dACC | 0.5 | 1000 | 36 | 1 |  | - | 1.5-5.38 | 3.5-4.5 | 0.2-0.57 | Inhibition | Both |
|  |  |  |  |  | 16.7 | 600 |  |  |  |  |  |
| Thalamus | 0.65 | 10 | 5, 70 | 30 | 50 | 720-1200 | 0.72-1 | - | - | Inhibition | Both |
| S1, S2, VPL | 0.21-0.5 | 300-1400 | 6-70 | 0.2-0.5 | - | - | 0.34-17.5 | 3-35 | 0.62-1.10 | Excitation | Both |
|  |  |  |  |  | 4.8 | 1280 |  |  |  |  |  |

Abbreviation: $f_{0}$: fundamental frequency; PRF: pulse repetition frequency; DC_pulse train_: duty cycle of a pulse train; PTD: pulse train duration; DC_pulse train repeat_: duty cycle of a pulse train repeat; PTRD: pulse train repeat duration; I_SPTA_: Intensity Spatial Peak Temporal Average; I_SPPA_: Intensity Spatial Peak Pulse Average; MI: mechanical index
