# Supplementary material for: Neuromodulation effects of low-intensity transcranial focused ultrasound in human, a systematic review focusing on motor and sensory functions
Source: J Neuroeng Rehabil. 2025 Nov 28;22:254. doi: 10.1186/s12984-025-01722-9 (PMC12664190; doi:10.1186/s12984-025-01722-9)
Supplement: Supplementary file 3 — Supplementary Material 3 [file 12984_2025_1722_MOESM3_ESM.docx]

**Supplementary Table 3. Summary of reported symptom rates in tFUS studies targeting motor and sensory function**

| Reported symptoms | Yu, 2021 (n=15) | Samuel, 2023 (n=10) | Ennasr, 2024 (n=18) | In, 2024 (n=16) | Legon, 2024 (n=23) | Strohman, 2024 (n=16) | Strohman, 2024 (n=16) | Zadeh, 2024 (n=21) | Average rate (%) |
| --- | --- | --- | --- | --- | --- | --- | --- | --- | --- |
| Scalp Sensations | 46.7 | 0 | 16.7 | 2.08 | 2.17 | 2.08 | 6.2 | 0 | 9.01 |
| Sleepiness | 0 | 0 | 16.7 | 6.25 | 4.35 | 16.67 | 0 | 9.52 | 7.17 |
| Neck Pain | 0 | 0 | 27.8 | 0 | 4.35 | 0 | 12.5 | 0 | 5.93 |
| Headache | 0 | 0 | 11.1 | 2.08 | 8.7 | 6.25 | 6.2 | 1.59 | 4.93 |
| Fatigue | 0 | 40 | 0 | 2.08 | 0 | 10.42 | 0 | 0 | 4.44 |
| Attention | 0 | 0 | 0 | 0 | 19.57 | 0 | 0 | 1.59 | 3.58 |
| Hand Symptoms | 0 | 0 | 5.6 | 4.17 | 0 | 2.08 | 0 | 11.11 | 3.22 |
| Nausea | 0 | 0 | 11.1 | 4.17 | 0 | 4.17 | 6.2 | 0 | 3.20 |
| Twitching | 0 | 0 | 5.6 | 6.25 | 0 | 4.17 | 0 | 6.35 | 2.97 |
| Tingling | 0 | 0 | 0 | 14.58 | 0 | 2.08 | 0 | 3.17 | 2.47 |
| Itchiness | 0 | 0 | 5.6 | 8.33 | 2.17 | 2.08 | 0 | 0 | 2.35 |
| Emotional | 0 | 0 | 0 | 4.17 | 0 | 10.42 | 0 | 1.59 | 1.98 |
| Unusual Sensations | 0 | 0 | 0 | 8.33 | 2.17 | 2.08 | 0 | 1.59 | 1.85 |
| Changes in Hearing | 0 | 0 | 0 | 4.17 | 2.17 | 0 | 0 | 3.17 | 1.36 |
| Reduced Coordination | 0 | 0 | 0 | 0 | 0 | 0 | 0 | 7.94 | 1.24 |
| Dizziness | 0 | 0 | 0 | 2.08 | 4.35 | 2.08 | 0 | 0 | 1.23 |
| Tooth Pain | 0 | 0 | 0 | 0 | 0 | 0 | 6.2 | 0 | 0.73 |
| Difficulty with Balance | 0 | 0 | 0 | 0 | 0 | 0 | 0 | 1.59 | 0.25 |
