# Supplementary material for: Neuromodulation effects of low-intensity transcranial focused ultrasound in human, a systematic review focusing on motor and sensory functions
Source: J Neuroeng Rehabil. 2025 Nov 28;22:254. doi: 10.1186/s12984-025-01722-9 (PMC12664190; doi:10.1186/s12984-025-01722-9)
Supplement: Supplementary file 4 — Supplementary Material 4 [file 12984_2025_1722_MOESM4_ESM.docx]

**Supplementary Table 4. Transcranial focused ultrasound studies on other brain regions.**

| Author | Targets | *f*_0_ (MHz) | PRF (Hz) | DC_pulse train_ (%) | PTD (s) | DC_pulse train repeat_ (%) | PTRD (s) | I_SPTA_ (W/cm^2^) | I_SPPA_  (W/cm^2^) | $p_{r}$(MPa) | MI | Main outcomes |
| --- | --- | --- | --- | --- | --- | --- | --- | --- | --- | --- | --- | --- |
| Lee, 2016 | Primary visual cortex | 0.27 | 500 | 50 | 0.3 | - | - | - | 16.6 | - | - | Elicited distinctive EEG peaks similar to VEPs. Having network activation. |
| Nakajima, 2022 | STN, anterior PFC, and anterior putamen | 0.5 | 10 | 30 | 40 | - | - | 9 | 30 | 0.495* | 0.70 | tFUS to STN and anterior putamen but not posterior putamen showed sustained disruption of response inhibition performance. tFUS to aIFC exhibited the sustained disruption of response inhibition performance significantly. tFUS suppressed MEPs. |
| Cain, 2021 | Left GP | 0.65 | 10 | 5 | 30 | 50 | 1200 | 0.72 | 14.39 | 1.06 | - | Decreased BOLD signal in the left GP. Reduced BOLD signal in cortical areas. Decreased perfusion in the left GP, putamen, and thalamus. |
| Butler, 2022 | Middle temporal complex | 0.5 | 1000 | 50 | 0.3 | - | - | - | - | - | - | Improved accuracy and reduced response times in visual motion detection tasks. |
| Sanguinetti, 2020 | rIFG | 0.5 | 40 | 0.26 | 30 | - | - | 0.13 | 16.2 | 1.27 | 1.8 | Decreased functional connectivity in rIFG and DMN. |
| Kim, 2022 | Medial PFC | 0.25 | 1400 | 70 | 0.3 | 9.1 | 1200 | - | - | 0.18 | 0.36 | Increased beta power and decreased theta power of EEG. |
| Fine, 2023 | rIFG | 0.5 | 1000 | 24 | 0.5 | - | - | 5.38 | 22.43 | 0.82 | 1.15 | Shortened the onset latency of the P300 event-related potential and reduced N200 amplitude. Improve response inhibition during stop signal. |
| Forster, 2023 | rIFG | 0.5 | 40 | 0.5 | 120 | - | - | 0.199 | 40 | - | 1.53 | tFUS altered alpha and theta activity at certain area. tFUS modulated neural activity but did not significantly alter the participants' subjective control perception. |
| Forster, 2023 | Right lateral PFC | 0.5 | 40 | 0.5 | 120 | - | - | 0.199 | 40 | - | 1.54 | Reduced theta power at Fz and Pz. Participants receiving tFUS demonstrated sustained task engagement despite the helpless condition. |
| Ziebell, 2023 | Right PFC | 0.5 | 40 | - | 1200 | - | - | - | - | 1.09 | - | Reduced MFT in conflict situations, especially during ambiguous events. No significant changes in self-reported mood. |
| Kuhn, 2023 | Amygdala or ErC | 0.65 | 100 | 5 | 30 | 50 | 300 | 0.72 | - | - | - | Increased cerebral perfusion and decreased BOLD activity in the amygdala. Reduced functional connectivity between the amygdala and functionally connected regions. Increased cerebral perfusion in the ErC. Increased functional connectivity between the ErC and left dorsolateral PFC. |
| Yaakub, 2023 | Cingulate cortex | 0.5 | 5 | 10 | 80 | - | - | 1.5 | 15.07 | 0.66 | - | tFUS targeted at PCC resulted in a significant reduction in GABA levels in the PCC voxel. Increased functional connectivity in both dACC and PCC, last at least 50 mins |
| Chou, 2024 | Amygdala | 0.65 | 10 | 5 | 30 | 50 | 1200 | 0.72 | 14.4 | - | - | Decreased BOLD in the amygdala during the fear task. Decreased amygdala-insula and amygdala-hippocampal rsFC. Increased amygdala and ventromedial PFC rsFC. |
| Chou, 2024 | VC/VS or ErC | 0.65 | 10 | 5 | 30 | 50 | 1200 | 0.72 | 14.4 | - | - | Increased putamen activation during reward task win trials. Enhanced rsFC between VC/VS and orbitofrontal cortex and anterior cingulate cortex. |
| Gorka, 2024 | Amygdala | 0.65 | 10 | 5 | 30 | 50 | 600 | 0.72 | - | - | - | tFUS targeting the amygdala significantly reduced connectivity between the salience network and frontoparietal network. tFUS targeting the ErC increased connectivity between the dorsal attention network and the DMN. |
|  | ErC |  | 100 |  |  |  |  |  |  |  |  |  |
| Hoang-Dang, 2024 | Right amygdala / Left ErC | 0.65 | 10, 100 | 5 | - | - | - | 0.72 | - | - | - | Increased self-reported arousal to negative images post-tFUS targeting the amygdala compared to the ErC. No significant changes in valence ratings for negative images and anxiety scores for either region. |
| Lord, 2024 | PCC | 0.5 | 10.562 | 5.26 | 30 | 50 | 300 | 0.024 | 0.46 | - | 0.18 | Reductions in functional connectivity along the midline of the DMN. Increased state mindfulness (sham too), reduced Global Vigor and other subjective effects. |
| Peng, 2024 | left NAc | 0.65 | 10 | 5 | 30 | 50 | 1200 | 0.995 | - | 0.72 | - | Inhibited activity in the bilateral NAc. Increased functional connectivity between the NAc and the medial PFC. |
| Kosnoff, 2024 | Visual motion-sensitive area V5 | 0.7 | 3000 | 60 | 0.5 | - | - | - | - | 0.2 | - | Reduced brain-computer interface speller errors. |

Abbreviation: VEP: visual evoked potential; STN: subthalamic nucleus; PFC: prefrontal cortex; GP: globus pallidus; BOLD: blood-oxygen-level-dependent; EMG: Electromyography; rIFG: right inferior frontal gyrus; DMN: default mode network; aIFC: anterior inferior frontal cortex; MFT: midfrontal theta activity; ErC: entorhinal cortex; PCC: posterior cingulate cortex; dACC: dorsal anterior cingulate cortex; rsFC: resting-state functional connectivity; VC/VS: ventral capsule/ventral striatum; NAc: nucleus accumbens
